# Determining the Community Prevalence of Acute Gastrointestinal Illness and Gaps in Surveillance of Acute Gastroenteritis and Foodborne Diseases in Guyana

**Published:** 2013-12

**Authors:** Shamdeo Persuad, Pheona Mohamed-Rambaran, Alexis Wilson, Colin James, Lisa Indar

**Affiliations:** ^1^Minsitry of Health, Guyana; ^2^Georgetown Public Hospital Corporation Laboratory, Ministry of Health, Guyana; ^3^Veterinary Public Health, Ministry of Health, Guyana; ^4^Caribbean Epidemiology Centre (CAREC/PAHO/WHO), Trinidad and Tobago

**Keywords:** Acute gastroenteritis, Burden of Illness Study, Diarrhoea, Guyana

## Abstract

Guyana is an English-speaking country in South America and, culturally, it is part of the Caribbean. Objective of this study was to determine the community prevalence and true burden and economic impact of acute gastroenteritis (AGE) and foodborne diseases (FBDs) in Guyana. A cross-sectional population-based survey was conducted in 7 of the 10 regions in Guyana during August and November 2009 to capture the high- and low-AGE season respectively. Overall, 1,254 individual surveys were administered at a response rate of 96.5%. The overall monthly prevalence of self-reported cases of AGE was 7.7% (97 cases) (95% CI 6.3-9.3), and the yearly incidence was 1.0 episodes per person-year. The highest monthly prevalence of AGE was observed in region 4 (8.9%) and in children aged 1-4 year(s) (12.7%). Of the 97 AGE cases, 23% sought medical care; 65% reported spending time at home due to their illness [range 1-20 day(s), mean 2.7 days], of whom 51% required other individuals to look after them while ill. The maximum number of stools per 24 hours ranged from 3 to 9 (mean 4.5), and number of days an individual suffered from AGE ranged from 1 to 21 day(s) (mean 2.7 days). The burden of syndromic AGE cases in the population for 2009 was estimated to be 131,012 cases compared to the reported 30,468 cases (76.7% underreporting), which implies that, for every syndromic case of AGE reported, there were additional 4.3 cases occurring in the community. For every laboratory-confirmed case of FBD/AGE pathogen reported, it was estimated that approximately 2,881 more cases were occurring in the population. *Giardia* was the most common foodborne pathogen isolated. The minimum estimated annual cost associated with the treatment for AGE was US$ 2,358,233.2, showing that AGE and FBD pose a huge economic burden on Guyana. Underreporting of AGE and foodborne pathogens, stool collection, and laboratory capacity were major gaps, affecting the surveillance of AGE in Guyana.

## INTRODUCTION

Guyana is an English-speaking country in South America and, culturally, it is part of the Caribbean. It is also one of the few Caribbean countries, which is not an island. The country has a total population of 751,225 and is divided into 10 regions, five of which are in the coastal areas, and four are inlands (1-2). Most (80%) people reside in the coastal lands in Region 2-6 (approximately 10% of the Guyana's land areas), and Region 4 is the most populated (with 41% of the total population). The population is predominantly rural, with only 30% of the population living in one of the 6 towns. The coastal areas are low-lying, crowded, and are prone to flooding and increased AGE-related illness during heavy rainfall. Cycles of flooding during December to February and May to June, with alternate rainfall and drought, have made sanitation difficult in these regions (1-5). There is one tertiary hospital and one reference laboratory. Agriculture is the main economic activity in Guyana, and over 80% of the population has access to potable water (2).

Acute gastroenteritis (AGE) is a major cause of morbidity worldwide, especially in children aged less than 5 years. Globally, one in every four children experiences at least 2 episodes of diarrhoea and vomiting; contaminated water, food, and poor hygiene have been identified as the major causes (6-10). In Guyana, the Ministry of Health has identified AGE as a significant public-health problem (3-5). The epidemiology of AGE and food- and waterborne illnesses at the community level is poorly understood as there is little information available on the disease incidence and burden relating to foodborne diseases (FBDs). Consequently, the already scarce resources available for disease control measures are not allocated towards appropriate prevention and control of food- and waterborne illnesses. The Ministry of Health reported that AGE occurs in every geographic region and that almost 30% of deaths among children below 5 years of age are caused by diarrhoeal illnesses (3). AGE occurrence is seasonal, and the highest incidence occurs in the months of December, January, February, and March (1,3,5)

The present system for the surveillance of communicable diseases in Guyana includes a weekly syndromic surveillance (reporting of AGE and other common syndromes) and four-weekly reports of laboratory-confirmed infectious diseases (including laboratory-confirmed AGE/FBD pathogens) to the Surveillance Unit of the Ministry of Health (1). These data indicated that there were 13,949 cases of AGE in 2007 and 32,634 cases in 2008. However, only 50 stool specimens from AGE cases were submitted during January 2007–September 2007 and, of these, only 4 cases of *Salmonella* were identified since the laboratory at that time was primarily testing for *Salmonella* (1). This suggests a potentially high burden of AGE and FBD, considering the small population-size and the low number of stool samples tested. However, the exact burden of AGE is unknown, and the proportion of AGE-related illness that is foodborne is not known since there are major problems of unreliable weekly reporting of AGE cases from all the regions and seldom collection of stools from AGE cases (3). In 2008, only 55% of the facilities reported consistently and on a timely basis. AGE is further underreported in the inland regions, which is characterized by marginalized populations, and many community-level outbreaks go unreported (1,3)

Sound information on disease burden of AGE and FBD is, thus, needed to guide the allocation of limited resources for appropriate intervention measures in Guyana. Many countries have conducted the World Health Organization (WHO)-recommended Burden of Illness studies to determine the true burden and impact of AGE and FBD in their populations (10-19). In 2009, the Ministry of Health of Guyana collaborated with the Caribbean Epidemiology Centre (CAREC) and the Pan American Health Organization (PAHO) to conduct a burden of AGE-related illness study in Guyana as part of the overall Caribbean Burden of Illness (BOI) Study. The objective of our study was to determine the community prevalence, true burden, and economic impact of AGE and FBD in Guyana. Additional technical and financial assistance was also provided by the Public Health Agency of Canada (PHAC) and the Caribbean Eco-Health Programme (CEHP).

## MATERIALS AND METHODS

### Population survey

A retrospective, cross-sectional population survey on AGE symptoms experienced by respondents in the 28 days prior to the interview was conducted by face-to-face interviews during 14-28 August 2009 and 15-28 November 2009 to account for variations in the high- and low-AGE season respectively. Seasonal variations were determined based on syndromic data collected for 2002 to 2007 by the Ministry of Health in Guyana. The survey was conducted in 7 of the 10 administrative regions of Guyana (Region 1-6 and 10). These 7 administrative regions were the most-populated regions, accounting for approximately 84% of the population. The other three regions were not easily accessible; hence, these were excluded. The WHO definition of diarrhoea was used in identifying self-reported cases of AGE: an acute (sudden) onset of diarrhoea with or without fever (>38 °C or 100.4 °F), presenting with three or more loose or watery stools within the past 24 hours with or without vomiting, and/or visible blood in stool (13).

### Sampling and selection of household and individuals

Based on 742,041 population in the 2002 Census, a sample-size of 1,279 was calculated using Epi Info (version 6.0) at an estimated prevalence of acute gastrointestinal illness (AGI) of 40%, a response rate of 80%, an allowable error of 3%, and a 95 % confidence interval. The final sample-size of 1,300 was used with 650 individual surveys conducted in each of the two periods (Phase 1 and 2).

Households were selected using a two-stage stratified random-sampling design. Data from the Bureau of Statistics Office (2000 Census) (1) were used in determining the number of Enumeration Districts (EDs), number of households per ED, and number of persons per household. The EDs were stratified by parish, and 1,300 samples were proportionately distributed among the EDs, based on the respective numbers of households. Houses to be sampled were randomly selected using a random number generator. An ED map was used for determining the start point and route for survey administration. Mentally-able persons aged more than 1 year and who were present in the country during the time of the survey were eligible to participate. Following these eligibility criteria, persons with their next birthdays falling before the interview date were selected.

Adults (mother, father, or primary caregiver) answered for eligible children aged less than 12 years. Adults were not allowed to serve as proxy for other adults. Written informed consent was obtained either directly from study participants or the parents, or primary caregivers of eligible children aged less than 18 years. Participants were not enrolled in the survey without a written consent. If an eligible adult or parent, or primary caregiver of a child aged below 18 years refused to either sign the consent form or to participate in the survey, the survey team member moved on to the next selected household. A maximum of three visits were made to each selected household before it was dropped, and the interviewers moved on to the next household. Each participant entering the survey was assigned a unique identification number that was used for ensuring confidentiality during the survey and data analysis. The identification number was included on the survey questionnaire and the consent form and was entered into the database. No incentives, monetary or others, were provided for participation in this survey.

### Data collection

Data were collected by trained interviewers via face-to-face interviews, using a standardized questionnaire developed by the Caribbean BOI Steering Committee. The questionnaire was pilot-tested in an electoral division that was not selected as part of the overall survey. Participants were asked if they experienced AGE symptoms in the 28 days prior to the interview and about the outcome of their illness. The survey also sought information on sociodemographics, frequency of healthcare-seeking, frequency of specimen submissions for appropriate laboratory tests, use of antibiotics and other medications, and perceived cause of illness. Completed data were stored at the Epidemiology Department of the Ministry of Health in locked cabinets accessible only by the coordinators. Sets of questionnaire were double-checked by the Nurse Epidemiologist to ensure that all relevant data were collected.

### Statistical analysis

Data were manually entered in EpiData (version 3.1) (EpiData Association Odense, Denmark). Each questionnaire was double-entered to reduce data-entry errors. Demographic characteristics were compared with those of the 2002 Census to determine the representativeness of the study population. Univariate and multivariate analyses were performed using Epi Info (version 3.1). Data were also analyzed to determine the response rate, characteristics of respondents, magnitude and distribution of diarrhoeal illness, severity of symptoms, medical care practices and patterns at 95% confidence interval. A p value of <0.05 was considered significant. Respondents aged less than 1 year and those staying outside the study regions were excluded. The sudden onset of diarrhoea (3 or more watery or loose stools within 24 hours with or without fever, vomiting, or visible blood in the stool) in the 4 weeks prior to the interview was classified as self-reported cases of AGE.

### Estimation of underreporting of AGE

**Figure 1.**
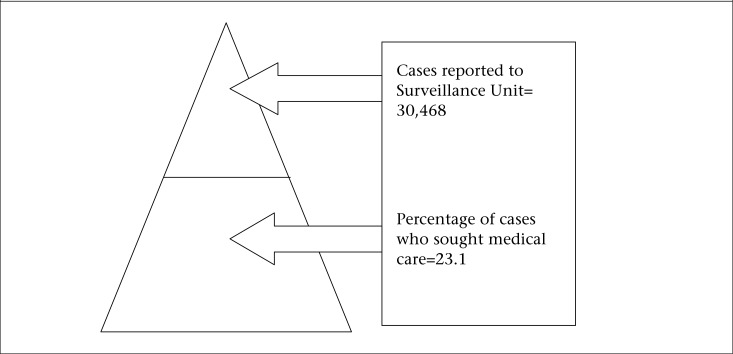
Estimation of underreporting and burden of syndromic acute gastrointestinal illness, Guyana

**Figure 2.**
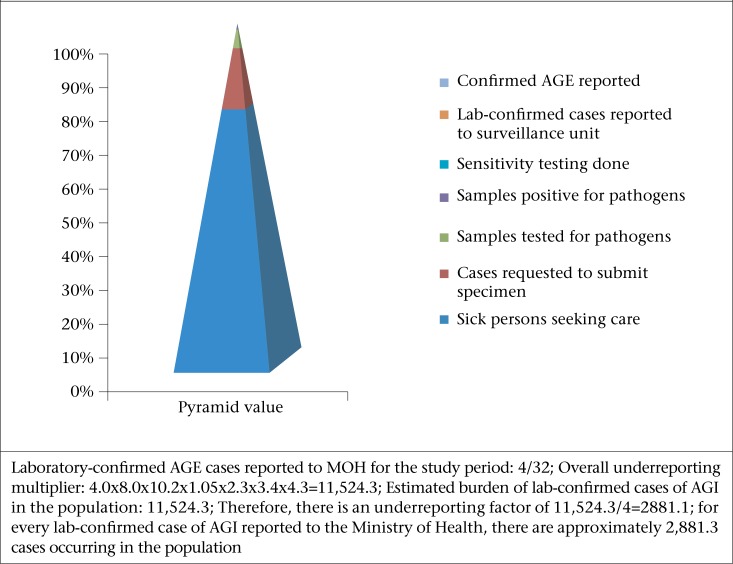
Estimation of underreporting and the burden of laboratory-confirmed acute gastrointestinal illness

Syndromic and laboratory-confirmed AGE data reported to the Ministry of Health Surveillance Unit for 2009 were compared with the data collected from the population-based survey and laboratory survey to calculate the underreporting and estimates of the burden of AGE in Guyana, based on the defined Burden of Illness pyramid for AGE surveillance in Guyana. The BOI pyramid was defined using the percentage of self-reported cases who sought medical care to estimate underreporting relative to syndromic AGE (Figure 1). The percentage of AGE cases who sought medical care submitted stool samples; stool samples were tested; samples testing positive for a foodborne pathogen and reported to the National Surveillance Unit were used in estimating underreporting relative to laboratory-confirmed AGE (Figure 2).

### Ethical approval

Ethical approval was granted from the Ministry of Health Ethical Board. All data collected were kept confidential. The names of the participants were not included in the questionnaire. Each participant was informed on the purpose of the survey and asked to sign a consent form before the questionnaire was administered.

### Laboratory survey

The laboratory survey was conducted for a period of one year—from January to December 2009—at the Georgetown Public Hospital Corporation Medical Laboratory, the only laboratory that conducts microbial analysis for FBD pathogens associated with AGE for both public and private health facilities in Guyana. Stool specimens submitted to the laboratory from patients with history of AGE were supposed to be tested for *Salmonella*, *Campylobacter*, *Shigella*, pathogenic *Escherichia coli*, *Staphylococcus aureus,* rotavirus, norovirus, and parasites (*Giardia and Cryptosporidium*). Data on the number of stool specimens received and tested in the laboratory, the proportion of cases lost to surveillance because of negative findings, and the proportion of confirmed cases reported to the surveillance systems were also collected.

### Estimation of economic burden of AGE

Estimates were calculated to reflect the potential economic burden on patients associated with accessing healthcare and treatment for AGE by multiplying the estimated episodes in the population per year by the minimum direct cost for basic medical services and treatment.

## RESULTS

### Response rate, characteristics, and representativeness of respondents

Of the total 1,300 individuals contacted to participate in the survey, 1,254 individual surveys were administered and completed: 607 in Phase 1 (14-28 August 2009) and 647 in Phase 2 (15-28 November 2009), with an overall response rate of 96.5% (Table 1). Fifty-five percent of the survey respondents were female, approximately 5% were aged 1-4 year(s), 17.1% were 5-14 years, 19.7% were 15-24 years, 26.6% were 25-44 years, 22.5% were 45-64 years, and 9.1% were ≥65 years of age. Comparison of the demographic profiles of residents in the general population and the survey respondents indicate that, on overall consideration, respondents were older than the census population and were more likely female (Table 1).

### Magnitude of illness

Of the 1,254 individual surveys completed, 97 respondents (7.7%) reported that they had sudden onset of diarrhoea in the 4 weeks prior to the interview and were, therefore, classified as self-reported cases of AGE. Thus, the overall monthly prevalence of AGE was 7.7% (95% CI 6.3-9.3), and the annual incidence was 1.0 episode per person-year.

**Figure 3.**
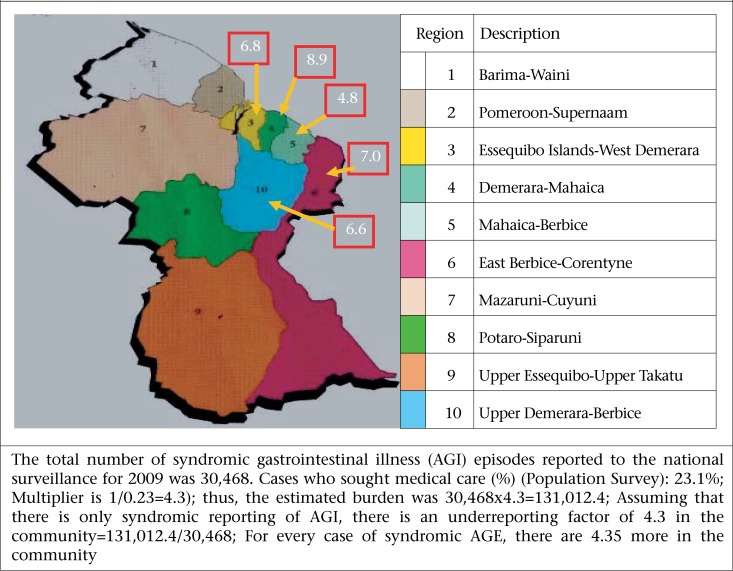
Prevalence of acute gastroenteritis in study regions in Guyana

The highest monthly prevalence of AGE was found in the 1-4 year(s) age-group (12.7%), followed by the 45-64 years age-group (9.2%); and the lowest reported prevalence of AGE was among persons aged 15-24 years (6.5%). These differences were not statistically significant. The age- and gender-adjusted monthly prevalence to population rates in Guyana were 7.61% and 7.68% respectively (Table 1). Prevalence of AGE also varied by region, with the highest monthly prevalence of self-reported cases of AGE reported in Region 4 (8.9 %), followed by region 6 (7.0 %) and Region 3 (6.8 %); the lowest reported monthly prevalence was in Region 5 (4.8%). This differences in the regions were statistically not significant (p=0.55) (Figure 3, Table 2). Overall, the monthly prevalence of AGE varied slightly between females (8.0%) and males (7.3%).

**Table 1.** Demographic characteristics of residents and survey respondents and monthly prevalence of acute self-reported gastrointestinal illness in Guyana

Gender and age	Survey cases			Population (%)	Gender/age-adjusted prevalence rate (%)
	No. ill	Respondents N (%)	Monthly prevalence rate (%)		
Gender					
Male	44	547 (44.4)	8.04	50.06	
Female	50	684 (55.6)	7.31	49.94	
Total	94	1,231			7.68
Age (in completed years)					
1-4	8	63 (5.0)	12.70	9.63	
5-14	18	214 (17.1)	8.41	23.90	
15-24	16	247 (19.7)	6.48	17.62	
25-44	23	334 (26.6)	6.89	29.26	
45-64	26	282 (22.5)	9.22	13.03	
≥65	5	114 (9.1)	4.39	0.35	
Total	96	1,254			7.61

### Symptoms and severity

Additional symptoms experienced by cases are outlined in Table 3. Of the 97 cases, more than half experienced abdominal pain (n=65), 42% reported headache (n=42) or nausea (n=39) while 30% experienced both vomiting and diarrhoea. The maximum number of stools per 24 hours ranged from 3 to 9, with a mean of 4.5 and a median of 4. The average number of days an individual suffered from AGI was 2.7, with a range of 1 to 21 day(s) and a median of 2 days. Of the 97 cases, 63 (65%) reported restricted activity and had to spend time at home due to their illness. The range of days spent at home due to illness was 1-20 day(s), with an average of 2.3 days and a median of 2 days. More than half (n=32) of the 97 cases required other individuals to look after them while ill. The range of days taking care of a case was 1-20 day(s), with an average of 3.9 days and a median of 2 days.

### Healthcare-seeking behaviour/Use of medical systems

Of the 97 cases, 22 (22.9%) sought medical care for their illness. Five visited an outpatient clinic of a private hospital, 9 attended an outpatient clinic of a public hospital, one attended a health centre, one went to a pharmacy, and 2 attended a private doctor's clinic. Two cases reported having been hospitalized. None reported that they visited a traditional healer or an alternative healthcare practitioner. Seven cases were requested for stool specimens, and all 7 reported they submitted their samples. However, only 3 reported that it was a stool sample while the others were urine samples (Table 4).

**Table 2.** Proportion of respondents and self-reported cases by regions in Guyana

Region	Population	High/Low- AGI season	Number of respondents (N=1,254)	Number of cases	Prevalence of AGI cases per health district (%)	Overall monthly prevalence of cases/region (%)
3	103,061	High	104	9	8.7	6.8
		Low	103	5	4.9	
4	31,0310	High	285	27	9.5	8.9
		Low	324	27	8.3	
5	52,428	High	52	2	3.8	4.8
		Low	53	3	5.7	
6	52,428	High	114	9	7.9	7.0
		Low	128	8	6.3	
10	41,114	High	52	3	5.8	6.6
		Low	39	3	7.7	

**Table 3.** Secondary symptoms, duration, and severity of symptoms in respondents with AGI in Guyana

Secondary symptom (No. of respondents)		No. of cases (%)		95% CI	
Fever (measured) (85)		11 (12.9)		6.6-22.0	
Fever (not measured) (92)		29 (31.5)		22.2-42.0	
Blood in stool (90)		6 (6.7)		2.5-13.9	
Vomiting (97)		29 (29.9)		21.0-40.0	
Abdominal pain (97)		65 (67.0)		56.7-76.2	
Headache (100)		42 (42)		32.2-52.3	
Nausea (94)		39 (41.5)		31.4-52.1	
Sore throat (94)		8 (8.5)		3.7-16.1	
Cough (94)		26 (27.7)		18.9-37.8	
Runny nose (96)		19 (19.8)		12.4-29.2	
Sneezing (96)		23 (24)		15.8-33.7	
Duration (days)	Mean		Median		Range
Duration of illness	2.7		2		1-21
Restricted to home (63)	2.3		2		0-20

### Estimation of underreporting and overall burden of AGE

Table 5 summarizes data of the population and laboratory surveys that would be used in calculating the burden and underreporting of syndromic AGE and laboratory-confirmed AGE/FBD pathogens in Guyana, using the BOI surveillance pyramids shown in Figure 1 and 2 respectively. Assuming that all the AGE cases who sought medical care in 2009 were reported to the Surveillance Unit of the Ministry of Health, the estimated burden of syndromic AGE cases in the population for 2009 was 131,012 compared to the reported 30,468 cases (Figure 1). This implied that, for every case of syndromic AGE reported to the MOH, there were additional 4.3 cases occurring in the community. Hence, the percentage of cases not reported is 76.7%. There is a greater degree of underreporting of laboratory-confirmed FBD/AGE-related pathogens. The estimated burden of laboratory-confirmed FBD/AGE-related pathogens in 2009 was 11,524 cases compared to the reported 4 cases. This showed an underreporting factor of 2881.1 (11,524.3/4), and an underreporting percentage of 99.9. Thus, for every laboratory-confirmed case of FBD/AGE-related pathogen, it was estimated that there were 2,881 cases occurring in the community.

### Socioeconomic costs

Using an estimated minimum cost of US$ 18 for treating an AGE case in Guyana [including direct cost of time (based on minimum wages) a health worker spends with AGE patient (US$ 4.00/patient) and average cost of basic consumables (ORS, pain killer, IV fluids, and antibiotics) associated with the treatment for AGE (US$ 14.00)], the annual economic burden of AGE was estimated to be US$ 23,582,332 (18×131,012 cases). Additionally, adults who were unable to perform routine activities lost, on average, between US$ 10 and 15 for each day that they were unable to work in, based on Guyana's minimum wages per day.

**Table 4.** Healthcare-seeking behaviour

Healthcare-seeking behaviour	Number of persons reporting	%	95% CI
Number of cases seeking medical care (97)	21	21.6	14.3-33.1
Number of cases asked to submit specimen (24)	7	29.2	12.6-51.1
Number submitting specimen (7)	7	100	-
Number taking antibiotics (6)	1	16.7	0.4-64.1
Number taking non-prescribed medications (89)	32	36.0	26.1-46.8

Median number of stools in 24 hours=4

**Table 5.** Data on laboratory testing, burden of AGI, Guyana, January 2009–December 2009

Information	Source of data	Formula and value
Number of syndromic AGE cases reported to the Ministry of Health	Ministry of Health Surveillance	30,468
Proportion of laboratory-confirmed AGE cases reported to MOH	Ministry of Health Surveillance	(4/32)=12.5%
Number of lab-confirmed AGE cases actually isolated at laboratory	Lab director (Lab survey)	32
Proportion of positive/laboratory-confirmed AGE (of AGE samples tested)	Lab director (Lab survey)	(32/328)=9.8%
Proportion of AGE samples tested at laboratory	Lab director (Lab survey)	(328/344)=95.3%
Proportion of AGE samples submitted to the laboratory	Population survey	(3/7)=42.9%
Proportion of AGE samples requested	Population survey	(7/21)=29.2%
Proportion of AGE cases seeking medical care	Population survey	(21/97)=23%
Number of AGE cases in population survey (meeting AGE case definition)	Population survey	97

## DISCUSSION

This is the first study to determine the prevalence and burden of AGE and FBD in Guyana. The study provided evidence to corroborate that AGE is a significant public-health burden and new evidence to indicate that the burden of AGE and FBD in Guyana is much higher than that reported to the Ministry of Health. The study also demonstrated that more than 75% of AGE cases were not reported and that infection due to *Giardia* was a common cause of AGE and food- and waterborne illnesses in this country. The study showed that AGE-related illness was posing a huge economic burden on Guyana at an estimated US$ 2 million per year. In addition to the gross underreporting of syndromic AGE cases and laboratory-confirmed FBD pathogens, the study also identified that inadequate surveillance of AGE, lack of stool collection from AGE cases, and inadequate laboratory capacity are major gaps in the surveillance system for AGE in Guyana. These gaps should, therefore, be addressed immediately to reduce diarrhoeal and FBD infections in Guyana. The results of the study are profound and will assist in the development of a national plan to control these preventable illnesses to formulate appropriate strategies for implementation by all stakeholders.

The results of our study confirm that diarrhoeal illness is common in Guyana, with approximately 7.7% of the population being affected annually at an incidence rate of 1 episode per person per year. The monthly prevalence of AGE (7.7%) (95% CI 6.3-9.3) identified in our study appears to be higher than that found in BOI studies conducted in other countries (12,14-16), indicating the public-health impact of this illness in this country. The high prevalence of AGE may be partly as a result of human geography, poor sanitation, and the use of unsafe non-potable water in Guyana. Most people live in rural areas and are crowded in the low-lying coastal plains (about 10% of Guyana's total land areas) where cycles of flooding and drought have historically made sanitation difficult. This is further exemplified in the inland areas where there is poor accessibility and an inconsistent supply of potable water. There are several large rivers running through the country, and accessibility to the inland areas often requires travel by boat or air (1,4) Unhygienic environmental conditions and unsafe water supply (use of non-potable untreated water from rivers and other sources) have been common contributing factors in several AGE, food- and waterborne diarrhoeal illness outbreaks in Guyana (2,4,5). In addition, the coastal plains have a hospitable environment for the malaria-carrying mosquitoes, and crowded housing on the plantations facilitates the spread of diseases. Sewage treatment remains inadequate in many rural households, especially in the village. More than 90% of the urban population and only 65% of the rural population had access to safe water in 1988. These factors will promote food- and waterborne parasitic infection (1,2,20). Consequently, the Ministry of Health had, in the past, implemented several environmental sanitation and hygiene programmes, especially in areas prone to flooding following droughts, as in Region 1 (1,2,5). Our findings of a high prevalence of AGE suggest the need to heighten the implementation of programmes on education, hygiene, and environmental sanitation, including ensuring the use of safe water (either potable or treated) in all communities in all regions in Guyana. Special programmes on hygiene, education, and sanitation should be made for certain target population, like natives living in the inland regions, who use the same river-water for cooking, bathing, fishing, transportation, and washing.

The finding of the estimated burden of AGE being substantially higher than that reported to Ministry of Health and the very high percentage of underreporting for syndromic AGE (76.6%) and laboratory-confirmed FBD/AGE pathogens (99.9%) imply that there are gaps in the surveillance system for AGE and other communicable diseases–from data collection to the reporting of illness to the Ministry of Health of Guyana–which could lead to policies and programmatic weaknesses to address public-health issues. Syndromic surveillance data collected by the Ministry of Health show inconsistent, incomplete and untimely weekly reporting of AGE cases from all regions (1). For example, in 2008, only 55% of the health facilities reported consistently and on a timely basis. Difficulty in accessibility to all the regions situated across the large land areas (sometimes requiring boat or air transport) further increases underreporting of AGE-related illness and hinders the timely transportation of stool specimens from many regions to the laboratory situated in Region 4. On many occasions, stool samples are not taken, and when taken, it often takes several days to weeks to reach the laboratory at which point the integrity of the samples received is often compromised (1,3). There is an urgent need to put measures in place to improve the surveillance of AGE-related illnesses in all regions. This should include measures to improve data and stool collection from AGE cases, timely and consistent analysis and reporting of these data, training of surveillance staff in all regions to conduct data analysis (to identify increases in a timely manner), and providing the necessary tools to conduct adequate surveillance and promotion of hygiene and sanitation (e.g. adequate supply of sterilizing agents for water) in all regions, especially in the regions that are difficult to access and do not have continuous access to potable water as in Region 1. Strengthening of regional laboratories to conduct basic food- and waterborne microbial testing, such as parasitic and faecal coliform tests, will promote early identification of causative organisms and prevention of FBD.

The lower response rate in Phase 1 compared to Phase 2 may have been due to the unavailability of the respondents during the study period which coincided with the week prior to resumption of school. This week usually have back-to-school activities nationally, such as shopping or enrollment at schools. However, the overall response rate was still high at 96.5% and could be accounted for by the system of using the closest house as replacement when a person was unavailable after two visits.

Comparison of the demographic profiles of residents in the general population and the survey respondents indicate that, overall, respondents were older than the 2002 Census population and were more likely female. This could likely be because females are more receptive to answering surveys, showing a possible bias in the population survey. The finding of an older population correlates with the declaration of the Ministry of Health of increased life-expectancy and suggests the need for an updated population census. The monthly prevalence of AGE was slightly higher in females (8.0%) than males (7.3%), with a gender-adjusted population prevalence of 7.68%. Although these differences were not statistically significant and could be purely artificial due to slightly higher number of females surveyed, this finding is similar to a previous study conducted in Canada where females were found to be more affected by AGE and may be associated with females having more exposure to contaminated products, such as meat and vegetables during preparation of meals (16,19).

The highest monthly prevalence of self-reported cases of AGE was reported in Region 4 (8.9 %) and the lowest in Region 5 (4.8%). Although the difference in the regions was statistically not significant (p=0.55), this finding was contrary to expectations since, among all the regions studied, Region 4 was the most accessible to potable water. The highest prevalence of AGE in Region 4 may, however, be confounded by the fact that this is the most densely-populated region and overcrowding and unsanitary conditions are common in some areas. The region has also a large number of small, makeshift food outlets; other confounders are the existence of many non-licensed food vendors and problems with sewage and other wastes as well as flooding. Additionally, data from our questionnaire which were not presented in the paper indicated that some of the AGE cases in this region did not always have access to potable water and/or bathed in rivers, both of which could have possibly contributed to AGE-related illness. Further analysis of these data is, thus, needed to determine if there is an association between the water supply and AGE-related illness.

The highest monthly prevalence of AGE was found in the 1-4 year(s) age-group (12.7%), followed by the 45-64 years age-group (9.2%); the lowest reported prevalence of AGE was found among persons aged 15-24 years (6.5%); these are similar to the findings from previous studies (11-16) and provide further evidence to corroborate that very young and the elderly are the vulnerable groups for AGE infections. This should guide policy and interventions on target groups for AGE.

The findings were not beyond expectation that more than half of the AGE cases experienced abdominal pain, over a quarter of the cases experienced vomiting (n=29), 42% of the cases reported headache (n=42) or nausea (n=39), and 30% experienced both vomiting and diarrhoea since these are all common secondary symptoms of AGE-related illness. However, over 60% of the persons with AGE were restricted to stay at home for their illness, of whom more than half (n=32) required other individuals to look after them while ill. This exerts an economic burden of AGE on the population of Guyana and could result in significant loss of productive days and costs to the national economy as well as the individuals.

The low proportion of self-reported cases who were asked to submit stool samples (7%) corroborated past data that indicate that stool collection from AGE cases is not frequently done in Guyana (I). This lack of stool collection and laboratory testing of stool samples of AGE cases severely hinders the determination of the causative agents of AGE-related illness in Guyana (whether parasitic, bacterial, viral, or chemical) and, thus, hinders the development of appropriate prevention and control measures for this important public-health problem. There is an urgent need to improve the surveillance system to monitor AGE and improve stool collection from AGE cases in Guyana.

The low yield of pathogenic organisms (9.9%) from specimens of patients affected with AGE and foodborne diseases could have been related to the lack of collection of diarrhoeal stools, inappropriate collection and transportation of specimens, lack of laboratory media, supplies, and capacity to test for the common FBD pathogens. It is well-known that samples are not normally stored at 4 °C and, sometimes, delivered 24 hours or later after collection. This delay in transportation may affect the viability of the causative organisms in the stool as well as promote the overgrowth of other organisms, making it difficult/impossible to isolate the causative organisms of the AGE-related illness. In addition, the lack of laboratory media allowed for testing all stools for *Salmonella*, *Shigella*, and parasites only, and not for the other common FBD pathogens (*Campylobacter, Vibrio,* rotavirus, and norovirus). Some samples were sent to CAREC for viral testing. Thus, the aetiology identified in our study was limited by the existing laboratory practices and capacity, the latter of which may have also contributed to the low yield of positive samples.

However, despite the limitations of the range of laboratory tests performed, the finding that *Giardia,* a parasitic FBD pathogen, was the most common pathogen isolated from the AGE stool samples is very instructive for guiding appropriate prevention and control measures for AGE and FBD infections in Guyana. Previous to this study, *Salmonella* bacteria were the most commonly-reported FBD pathogen (22). Giardiasis is an illness caused by a parasite called *Giardia intestinalis,* infecting the small intestine. It lives in soil, food, and water. It may also be on surfaces that have been contaminated with wastes. Giardiasis is contracted by drinking water from contaminated lakes or streams and can also spread by direct person-to-person contact, which has caused outbreaks in institutions, such as day-care centres. One can also get it if exposed to human faeces (poop) through sexual contact. Exposure of a family member to giardiasis and unprotected anal sex are common risk factors (20). Conditions of overcrowding, cycles of drought and flooding, poor hygiene and sanitation and the use of non-potable water in some of the regions (especially the coastal regions) in Guyana may have all contributed to the isolation of *Giardia* as the most common cause of AGE in our study. The best way to prevent *Giardia* infection is to practise good hygiene, including frequent handwashing, drinking potable safe water, and also peeling or washing fresh fruits and vegetables before eating (21).

In Guyana, *Giardia* tests are not routinely performed, nor is it routinely reported to the Ministry of Health in Guyana. Given that hygiene and sanitation conditions in the regions, flooding, and the use of non-potable unsafe water in the regions are known common factors to promote *Giardia* infections and that this parasite was the most common pathogen isolated, we strongly recommend that the capacities of all laboratories in all 10 regions be strengthened to test for *Giardia*, report this illness in a timely manner to the Ministry of Health. Hygiene, sanitation, and the use of safe water (sterilization of river-water) should be ensured.

This study also highlighted the following gaps in the national surveillance system for AGE in Guyana: (i) Untimely and incomplete reporting of laboratory data to the Ministry of Health: The Ministry of Health did not receive regular reports on pathogens isolated from AGE specimens at the laboratory. Therefore, such information is not available for use in planning. Although a system was instituted to report on isolated pathogens at the laboratory, several irregularities continue to exist in proper labelling of samples from patients with a history of diarrhoea at the intake point, and there exist gaps in the timely reports submitted to the Ministry. Additionally, reports were not submitted on *Giardia*; (ii) Infrequent request and collection of stool specimens: Despite sensitization efforts to encourage physicians to request for stool samples from patients with symptoms of diarrhoeal illness, it was reported that many patients were treated without a request for stool samples. The small number of stool samples presented to the laboratory for testing has largely contributed to limited information about common foodborne pathogens in Guyana and underreporting of confirmed AGE cases to the national surveillance; (iii) Untimely transportation of specimens to laboratory: The lack of regular transportation system from the health centres and the absence of bacteriological testing at regional laboratories resulted in a situation that specimens often reach the laboratory later than the recommended period of less than 4 hours. Samples are also stored and transported at room temperatures to the laboratory; (iv) Delays in between the receipt of samples and reporting of results: Since a regular schedule does not exist to produce reports for patients, time to hand over the laboratory test results to patients can vary between one and four weeks, during which time a patient might be already treated and had recovered; and (v) Untimely dissemination of results to epidemiology unit and the use of results for action: While improvements were made in the frequency of reporting during the study period, some pathogens were not reported, and reports were not submitted regularly. Results from laboratory findings are not usually used for informing specific/heightened public awareness campaigns or special responses.

Finally, the estimated economic cost of treating syndromic AGE-related illness in Guyana (over US$ 2 million) is alarming, especially for a developing country, like Guyana. This was the minimum cost and, in fact, will be much higher for treating severe AGE cases and for laboratory-confirmed FBD pathogens. These data are very instructive for the policy-makers, showing ample justification for putting funds into food safety and foodborne illness prevention measures (for example, strengthening the surveillance of AGE and FBD, including laboratory capacity, and improving hygiene and sanitation in the regions), rather than treating AGE-related illnesses.

### Conclusions

To improve AGE and FBD surveillance in Guyana and thereby reduce the burden of FBD infections, we propose the following priority actions and interventions: (i) Enhanced surveillance of AGE and FBD, including stool collection, detection of pathogens, timely notification, reporting, and investigation of outbreaks over the next 12 months; (ii) Training and implementation of testing for *Giardia* and other protozoa from AGE stool specimens in all the regional laboratories and implementing norovirus testing at the reference laboratory; (iii) Implementation of measures to ensure timely and complete four-weekly reporting of laboratory data to the Ministry of Health and provision of timely feedback to clinicians, environmental health and laboratory personnel on reported AGE over four weeks; and (iv) Training and updating of all health workers and other stakeholders on the relevant reporting systems and AGE investigation over the next two years.

## ACKNOWLEDGEMENTS

The authors would like to thank all who contributed to making this study possible. Special thanks to CAREC/PAHO and PHAC for driving the process and to IDRC and the Caribbean Eco-Health Programme for assisting with funding. Thanks to Sarah Quesnel of CAREC, who facilitated the analysis of data. Special thanks are also due to Dr. Leslie Ramsammy, Minister of Health; the surveillance staff at the Ministry of Health, the laboratory; interviewers; Bureau of Statistics; and the BOI study team for coordination. Lastly, we must thank the respondents for their participation.
